# 3D ^7^Li magnetic resonance imaging of brain lithium distribution in bipolar disorder

**DOI:** 10.1038/s41380-018-0016-6

**Published:** 2018-02-09

**Authors:** Fiona Elizabeth Smith, Peter Edward Thelwall, Joe Necus, Carly Jay Flowers, Andrew Matthew Blamire, David Andrew Cousins

**Affiliations:** 1 Institute of Cellular Medicine, Newcastle University, Newcastle upon Tyne, NE1 7RU UK; 2 Newcastle Magnetic Resonance Centre, Campus for Ageing and Vitality, Newcastle University, Newcastle upon Tyne, NE4 5PL UK; 3 Institute of Neuroscience, Newcastle University, Newcastle upon Tyne, NE1 7RU UK

## Abstract

Lithium is a major treatment for bipolar disorder and the likelihood of a favourable response may be determined by its distribution in the brain. Lithium can be directly detected by magnetic resonance (MR), but previous ^7^Li MR spectroscopy studies have demonstrated that this is challenging compared to conventional ^1^H MR imaging due to the MR properties of the lithium nucleus and its low concentration in brain tissue, as dictated by therapeutic dose. We have tested and implemented a highly efficient balanced steady-state free precession ^7^Li-MRI method to address these challenges and enable MRI of brain lithium in a short duration scan. We report a 3D ^7^Li-MRI acquisition with 25 mm isotropic resolution in an 8-min scan that demonstrates heterogeneity in lithium concentration within the brain in subjects with bipolar disorder. This represents the direct imaging of a pharmaceutical agent in its target organ and notably expands the repertoire of techniques available to investigate the effects of lithium in man.

## Introduction

Lithium has long been prescribed for the affective disorders and retains a dominant position in major treatment guidelines for bipolar disorder [1]. While much is known about the cellular effects of lithium [2], our understanding of its distribution in the brain remains in its infancy [3], and this disparity hinders the interpretation of key findings. For instance, lithium-related changes in neurometabolite concentration varies by brain region [4], but it is not known whether this reflects differences in lobar sensitivity to lithium or lobar variations in lithium concentration. It has reasonably been proposed that the efficacy of lithium might in some way be related to its distribution in the brain [5]—were it to prove non-uniform, identifying patterns of distribution might permit the discrimination of responders from non-responders and lead to the stratification of treatment.

Lithium-7 (^7^Li), the most abundant stable isotope of lithium, can be detected by magnetic resonance [6] (MR), but it has lower MR sensitivity than the ^1^H nuclei detected in conventional magnetic resonance imaging (MRI). The low tissue concentration achieved in the therapeutic range also results in low signal-to-noise ratios (SNRs) [7] with brain lithium MR techniques, and the long T_1_ relaxation time of ^7^Li in vivo [8] has hindered rapid data acquisition. While advances in MR detection of ^7^Li have recently been reported [5, 8–11], these approaches have employed either localised MR spectroscopy or spectroscopic imaging (MRSI) techniques. MR spectroscopy acquires data in such a way to specifically discriminate between (and create images of) multiple MR resonances, typically originating from different chemical species within a tissue—for example, ^1^H MRSI has been widely used to image brain metabolite distribution. However, for in vivo ^7^Li MR only one chemical species is observed, and the resonance of ^7^Li is a spectral singlet. Thus there is no barrier to application of methodologies [12] used in conventional ^1^H-MRI instead of time-inefficient MRSI or to the use of advanced MRI techniques to maximise image quality while minimising scan duration. One such approach is balanced steady-state free precession (b-SSFP), which is well suited to detection of species with long T_1_ and in applications where low SNR impedes data acquisition. We hypothesised that application of b-SSFP methods to ^7^Li-MRI would afford considerable gains in image SNR compared to conventional spoiled gradient echo (SPGR) imaging and MRSI methods and thus permit assessment of brain lithium distribution in a scan with a clinically acceptable duration.

## Methods

### Subjects and assessments

Twelve euthymic subjects with bipolar disorder recruited to the Bipolar Lithium Imaging and Spectroscopy Study underwent ^7^Li-MRI. All subjects provided written informed consent, and the study was granted a favourable ethical opinion by a United Kingdom National Research Ethics Committee (14/NE/1135). Subjects were eligible for inclusion if they had a diagnosis of bipolar disorder (I or II) and were taking lithium as a long-term treatment. Exclusion criteria were contraindications to MR examination, current or recent harmful drug or alcohol use, comorbid diagnosis, learning disability, impairment of capacity or current liability to detention under the Mental Health Act 1983 (amended 2007). Diagnosis was confirmed through clinical interview using the NetSCID diagnostic tool (a validated online version of the Structured Clinical Interview for Diagnostic and Statistical Manual of Mental Disorders, Fifth Edition Criteria [13]; Telesage, Inc., Chapel Hill, NC, USA). All interviews and objective ratings were conducted by the same researcher (CJF) and discussed with a senior psychiatrist (DAC). Euthymic mood state was confirmed at entry to the study, defined as scores of <7 on both the 21-item Hamilton Depression Rating Scale [14] and the Young Mania Rating Scale [15]. All subjects had taken lithium carbonate (Priadel™ modified release once daily) regularly for at least 1 year at the time of recruitment and all were taking at least one concomitant medication for the treatment of bipolar disorder. Response to lithium was determined using the Retrospective Evaluation of Lithium Response (Alda scale) [16] and non-target effects were gauged using the Lithium Side Effects Rating Scale (LISERS) [17]. Scans were performed at 9 a.m. and subjects were instructed to take their lithium as usual the night before. Blood samples were taken immediately prior to scanning and serum lithium concentration was measured by colourimetric assay on a Roche Cobas 702 analyser.

### Scanner and coil system

MR data were acquired on a Philips 3 T Achieva scanner (Philips Medical Systems, Best, The Netherlands) with multinuclear capability and equipped with a quadrature, double-tuned ^1^H/^7^Li radiofrequency (RF) birdcage head coil (RAPID Biomedical, Rimpar, Germany).

### ^7^Li 3D b-SSFP gradient echo imaging

A ^7^Li 3D b-SSFP gradient echo acquisition was acquired in eight subjects using a protocol based on the in vivo relaxation (T_1_ and T_2_) properties of ^7^Li (20 × 19 × 7 acquisition matrix and 24.0 × 25.3 × 25.0 voxel size, 480 × 480 × 175 mm^3^ field of view, with TR = 9.4 ms, TE = 4.5 ms, flip angle = 60 degrees, receiver bandwidth = 219 Hz/pixel, 500 averages per dynamic with a standard elliptical *k*-space under-sampling scheme [18]). Data were reconstructed into a 32 × 32 × 7 matrix with a voxel size of 15 × 15 × 25 mm^3^. Total acquisition time was 8 min.

The scan protocol was also applied to test objects containing lithium to evaluate image uniformity and the relationship between lithium concentration and ^7^Li signal intensity in b-SSFP images. Six 0.8 L aqueous phantoms were constructed, each containing a different concentration of LiCl (0.5, 0.75, 1.0, 1.5, 2.0 and 4.0 mmol/L) and NaCl at a concentration of 108 mmol/L.

### ^7^Li 3D SPGR imaging

A further four subjects underwent an extended scan protocol in which ^7^Li 3D SPGR images (i.e., standard gradient echo imaging) were acquired in addition to ^7^Li b-SSFP data for comparison of image SNR between the two techniques. The ^7^Li SPGR imaging sequence was acquired with the same acquisition matrix size, field of view, reconstruction matrix and scan duration as the ^7^Li b-SSFP acquisition. Other parameters were set to maximise SNR (TR = 6.7 and TE = 2 ms, flip angle was the Ernst angle at 4.6 degrees, 700 averages per dynamic and a standard elliptical *k*-space under-sampling scheme). Each acquisition was 8 min long, and to increase image SNR, three scan dynamics were acquired in each subject (total acquisition 24 min) and the data averaged in complex form. Comparably, three ^7^Li b-SSFP dynamics were acquired in each subject (total acquisition 24 min) and similarly averaged in complex form.

### 3D T_1_-weighted imaging

A 3D T_1_-weighted image of brain anatomy was acquired for each subject with a ^1^H gradient echo sequence (TR = 8.2 ms, TE = 4.6 ms, acquisition matrix = 180 × 200 × 146 mm^3^ reconstructed into a matrix size of 240 × 240 × 146 mm^3^, isotropic 1 mm resolution, field of view = 216 × 240 × 175, one average).

### Image processing and analysis

#### ^7^Li-MRI preprocessing

All reconstructed lithium images were exported in DICOM format and converted to Analyze format using MRIconvert version 2.0 Rev. 235 for Windows (Lewis Center for Neuroimaging, University of Oregon, OR, USA). The images were then interpolated (bilinear) to match the resolution parameters of the ^1^H MRI using in-house software written in Matlab^®^ (The Mathworks^®^ Inc., Natick, MA, USA).

#### ^7^Li-MRI analysis

^7^Li images were analysed using the ImageJ software [19] and in-house software written in Matlab. To determine the relationship between ^7^Li-MRI signal intensity and lithium concentration, a region of interest (ROI) was placed in the central image slice of each test object. To evaluate the spatial distribution of ^7^Li-MRI signal intensity, a volume ROI (40 × 40 × 40 mm) was placed in each test object image and the coefficient of variation (COV) was measured.

The spatial distribution of ^7^Li b-SSFP signal intensity in subjects was evaluated by the measurement of the mean within-slice COV. For each subject and each ^7^Li b-SSFP axial slice, the ^1^H MRI axial slice that most closely aligned with the middle of the ^7^Li b-SSFP slice in the z axis was identified. An intracranial ROI was drawn on each ^1^H MRI axial slice in native space and applied to the ^7^Li b-SSFP image slice. The COV in signal intensity was averaged across slices and then for all subjects (*n* = 8). For the comparison of signal intensity between the ^7^Li b-SSFP and SPGR sequences, mean ^7^Li MRI signal amplitude was also determined from intracranial volume ROI.

#### Tissue-class ROI analysis

Individual ^1^H MR images were registered to the standard Montreal Neurological Institute (MNI) 2 mm brain template using FMRIB’s Non Linear Image Registration Tool [20]. For each subject, interpolated ^7^Li b-SSFP images were transformed into MNI space by applying the transformation matrix obtained from registering their ^1^H MRI to standard space. Mean ^7^Li b-SSFP signal intensity was measured for each subject within defined ROI for whole grey matter (GM; Harvard-Oxford cortical 2 mm atlas) and whole white matter (WM; John Hopkins University 2 mm WM atlas).

For the purpose of visualisation of mean signal intensity distribution, a group-wise average lithium image was generated using the standard space normalised ^7^Li b-SSFP images.

#### Statistical analysis

Descriptive and comparative statistics were conducted in SPSS Version 24.0, with data presented as mean ± standard deviation unless otherwise stated. For the comparative statistics, data met the assumptions for normal distribution and so parametric tests were applied. In reporting the development of an imaging technique, an a priori power calculation for sample size was not possible.

## Results

### ^7^Li b-SSFP gradient echo imaging

#### Test object studies

**Fig. 1:**
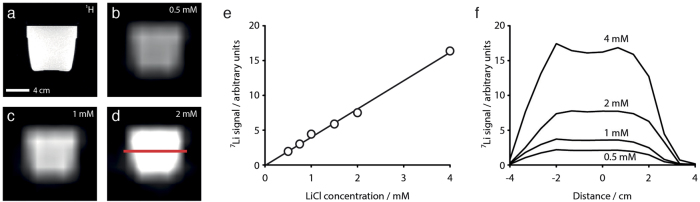
^7^Li b-SSFP MRI in test objects: **a** conventional ^1^H MRI; **b** ^7^Li b-SSFP MRI at LiCl 0.5 mmol/L; **c** ^7^Li b-SSFP MRI at LiCl 1.0 mmol/L; **d** ^7^Li b-SSFP MRI at LiCl 2.0 mmol/L; **e** correlation between ^7^Li b-SSFP MRI signal intensity and LiCl concentration; **f** uniformity of ^7^Li b-SSFP MRI signal intensity across the test object images

Analysis of the images of the six uniform test objects containing 0.5–4 mmol/L LiCl demonstrated a linear relationship between lithium concentration and ^7^Li b-SSFP image intensity. ^7^Li b-SSFP images from four test objects and the corresponding conventional ^1^H MRI are shown in Fig. 1a–d. Figure 1e shows the linear correlation (Pearson coefficient 0.98) between ^7^Li b-SSFP signal intensity and lithium concentration for these test objects. ^7^Li b-SSFP signal intensity profiles through the same test objects (measured at the level marked by the red line in Fig. 1d) are shown in Fig. 1f and demonstrate uniformity of ^7^Li b-SSFP signal intensity across the images. Further, the COV was determined to be 2.6 ± 0.3%, confirming that the ^7^Li b-SSFP sequence generated uniform signal distribution over the volume of the uniform test objects.

#### Human subject studies

The individual characteristics of the eight subjects (4 F/4 M; mean age 44 ± 13.6 years) who underwent ^7^Li b-SSFP are presented in Table 1. All subjects were taking lithium as a maintenance treatment (range 1–15 years, mean 6.4 ± 5.4 years); the mean once-daily dose of Priadel™ was 1000 mg ± 151 mg and the average serum lithium concentration was 0.88 ± 0.12 mmol/L.

**Table 1:** Individual characteristics and region of interest signal intensity for the eight subjects with bipolar disorder who underwent ^7^Li b-SSFP MRI

Subject	Age (years)	Sex	Duration of treatment (years)	Priadel™ dose (mg)	Serum lithium concentration (mmol/L)	Alda scale scores			LISERS scores	^7^Li b-SSFP signal intensity (arbitrary units)	
						Total score	A	B		White matter	Grey matter
A	57	M	15	1200	1.0	9	10	1	6	6.25	4.98
B	32	F	2	1000	1.0	1	6	5	13	7.45	5.76
C	48	F	8	1000	1.0	2	4	2	18	5.25	4.43
D	21	M	2	1000	0.9	4	7	3	25	4.76	4.76
E	52	F	9	1000	0.8	3	5	2	48	6.67	5.71
F	59	M	12	800	0.8	2	4	2	16	5.81	5.36
G	50	M	2	1200	0.8	1	3	2	35	6.92	5.56
H	33	F	1	800	0.7	5	8	3	21	5.72	5.23
									Mean	6.10	5.22

**Fig. 2:**
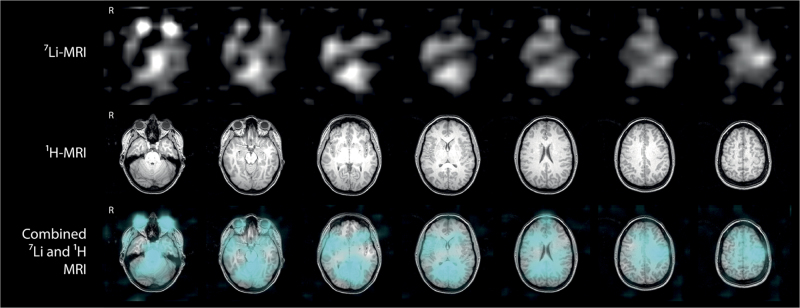
^7^Li b-SSFP MRI in a single subject. The ^7^Li-MRI dataset (upper row) was acquired in 8 min and is presented with corresponding ^1^H-MRI axial images (middle row). The combined images (lower row) show ^7^Li b-SSFP MRI (in cyan) overlaid on ^1^H-MRI images. The distribution of lithium in the brain is heterogeneous and the greatest signal intensity arises from the orbits

**Fig. 3:**
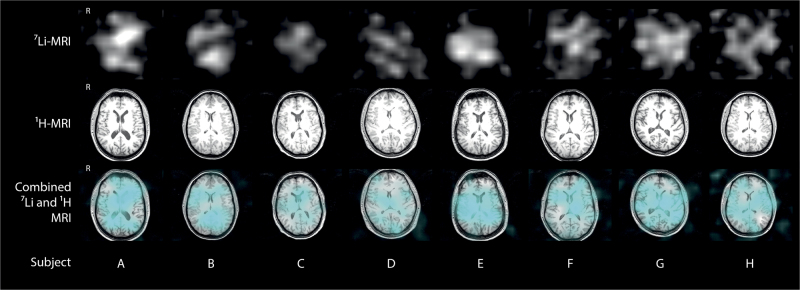
^7^Li b-SSFP MRI in eight subjects shown at the level of the head of the caudate nucleus (upper row), with corresponding ^1^H-MRI axial slices (middle row). The combined images (lower row) show ^7^Li b-SSFP MRI (in cyan) overlaid on ^1^H-MRI images. The distribution of lithium in the brain is heterogeneous in all subjects and varies between subjects

Heterogeneity of lithium distribution across the brain was observed in all subjects. Figure 2 shows ^7^Li b-SSFP images and corresponding ^1^H MRI axial slices from one individual (subject B), demonstrating heterogeneous distribution of lithium throughout the brain. Figure 3 shows individual ^7^Li b-SSFP images and the corresponding ^1^H MRI axial slice at the level of the head of the caudate nucleus from each of the eight subjects, with the distribution seen to differ between subjects. The average COV of ^7^Li b-SSFP signal intensity was 27.9 ± 3.6%, numerically confirming that brain lithium distribution is heterogeneous.

**Fig. 4:**
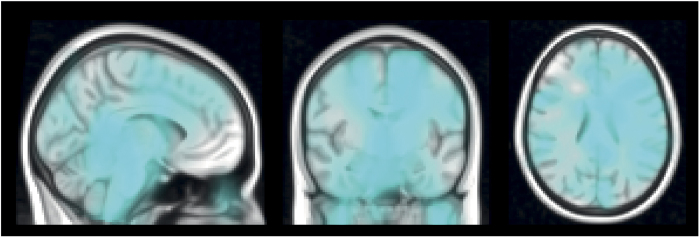
Group-wise average ^7^Li b-SSFP MRI. Standard space normalised ^7^Li b-SSFP images of eight subjects averaged and overlaid on MNI standard brain

In the tissue-class ROI analysis, ^7^Li b-SSFP mean signal intensity was significantly greater in WM compared to GM (6.10 ± 0.89 versus 5.22 ± 0.47 versus; arbitrary units, *T* = 2.46, *p* = 0.03). Serum lithium concentration, LISERS scores and Alda scale scores did not correlate with ^7^Li b-SSFP signal intensity in either whole WM or whole GM. The group average ^7^Li b-SSFP image (Fig. 4) demonstrated particularly high signal intensity in the brainstem—a post-hoc analysis placing a spherical ROI in this area revealed no correlation between ^7^Li b-SSFP signal intensity and serum lithium concentration, LISERS scores or Alda scale scores.

#### ^7^Li SPGR imaging

**Fig. 5:**
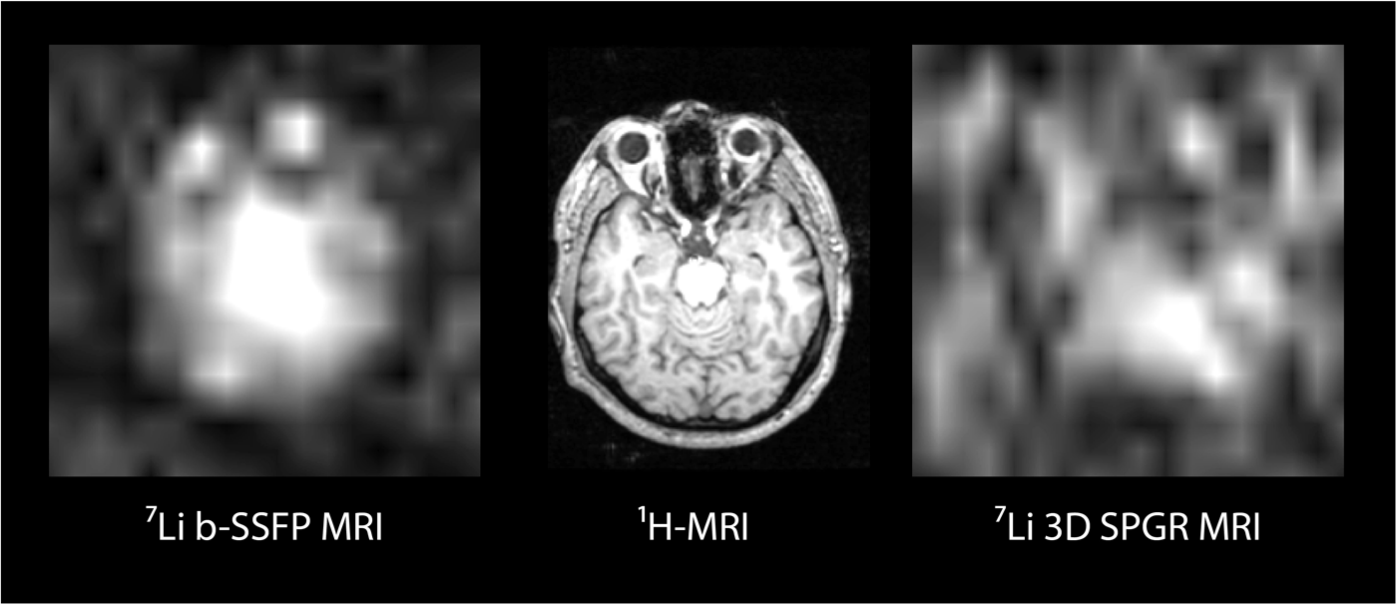
Comparison of the ^7^Li b-SSFP MRI and ^7^Li SPGR MRI in a single subject, plotted with the same intensity scale and acquired with equal scan durations (24 min in total for each sequence)

^7^Li b-SSFP MRI produced images with notably higher SNR than the comparable ^7^Li SPGR MRI in the four subjects who underwent the extended scan protocol that compared these two acquisition sequences. The ^7^Li SPGR imaging sequence failed to produce images with adequate SNR for visualisation of brain ^7^Li distribution in three out of the four subjects, despite a 24 min scan duration (three times the duration of ^7^Li b-SSFP scans shown in Figs. 2 and 3). All ^7^Li b-SSFP images showed brain ^7^Li signal amplitudes markedly greater than image noise levels. The ^7^Li SPGR scan in the one subject with sufficient SNR for comparison with ^7^Li b-SSFP is shown in Fig. 5—the ^7^Li b-SSFP and ^7^Li SPGR images are plotted with the same intensity scale and acquired with equal scan durations (both 24 min in total). In an ROI analysis, signal intensity in the brain was three times higher in the ^7^Li b-SSFP images than in the ^7^Li spoiled gradient echo images. We performed modelling to examine the theoretical performance benefit of the ^7^Li b-SSFP sequence (supplementary information), which indicated that an enhancement of at least a factor of two over spoiled gradient echo would be expected, consistent with this measured improvement.

## Discussion

Our data demonstrate the ability of ^7^Li b-SSFP MRI to rapidly image lithium in the human brain in vivo and present evidence of heterogeneous distribution. The use of an imaging-based acquisition rather than a spectroscopic imaging approach, coupled with the time- and signal-efficient steady-state free precession methods, affords considerable advantages compared to previous reports [5, 10, 11] and makes the measurement of brain lithium distribution practical for more widespread application in clinical research. Our development of this technique on a 3 T clinical scanner rather than a high-field research scanner further enhances the potential for a broad adoption of this method.

There is a lack of good quality post-mortem data on the brain distribution of lithium in patients with bipolar disorder [21, 22]. Preclinical rodent tissue studies largely report a non-uniform distribution of lithium in the brain following acute administration [23–31], with mixed findings of even [32, 33] and uneven [28, 29, 34] distribution after chronic administration. Many of these studies have relied on relatively crude tissue homogenization atomic absorption techniques and a consensus on the distribution profile has not been reached. A recent, high-resolution time-of-flight secondary ion mass spectroscopy study on the extracted rat brain reported relatively high concentrations of lithium in the olfactory bulb, hippocampus and subventricular zone, interpreted as consistent with a neurorestorative action of lithium [35]. Rodent MRSI studies have also reported regional variation in ^7^Li signal intensity across the brain in vivo [36, 37], but to achieve adequate resolution, scan duration is often several hours such that pharmacokinetic factors must be considered when interpreting signal intensity [38–40]. A recent ultra-high field strength, ex vivo ^7^Li-MRI study of the rat brain also reported signal heterogeneity as well an overall correlation between signal intensity and mass spectroscopy-derived lithium concentrations across brain regions [41]. We observed heterogeneity in lithium distribution in all subjects, but visually different distributions between subjects. Averaged data from normalized images demonstrated higher signal intensity in WM compared to GM. Notably, areas of high signal intensity included the brainstem and posterior corpus callosum, with low intensity in the orbitofrontal cortex. A consistent observation was high signal intensity arising from the orbits. Our ^7^Li-MRI technique does not have the resolution to determine the exact origin of this orbital signal, but it is interesting to note that rodent studies have demonstrated that lithium is concentrated in the retina, exceeding plasma concentrations in high- and low-dose regimes [42]. Our phantom studies clearly demonstrate uniform ^7^Li image intensity in objects with a homogeneous distribution of lithium. ^7^Li signal amplitude in our datasets is determined by lithium concentration and the MR relaxation properties (T_1_ and T_2_) of the ^7^Li nucleus. Were the T_1_ and T_2_ to vary in vivo across the brain, this might contribute to the observed heterogeneity. However, modelling of the dependence of b-SSFP signal amplitude on ^7^Li T_1_ and T_2_ (see supplementary information) gives us confidence that the spatially heterogeneous distribution of the ^7^Li b-SSFP signal observed in the human brain reflects heterogeneity in lithium tissue concentration. This agrees with preclinical mass spectrometry-based brain lithium quantitation study that also reported heterogeneity of lithium concentration between sampled tissues [35]. Nevertheless, future studies may wish to measure regional ^7^Li T_1_ and T_2_ relaxation times to explore this area in greater detail.

Previous studies have variously demonstrated moderate correlations between brain lithium concentration and serum concentrations [43], as well as treatment response and side effects [44, 45]. We did not find such correlations, but in our subjects, the serum concentration of lithium was very tightly within the therapeutic range, unlike the other reports. Furthermore, the heterogeneity observed in brain ^7^Li-MRI signal distribution questions the validity of correlating whole-brain lithium concentration against measures such as serum concentration or broad assessments of side effects. The relationship between high ^7^Li-MRI signal intensity in the brainstem and specific side effects such as ataxia and tremor is an area for consideration in future studies.

The basis of the heterogeneity in lithium distribution requires further exploration. Factors such as age, sex, duration of treatment and response (which were quite varied in our sample) could conceivably contribute to differences in distribution between subjects. Investigation of healthy subjects given lithium would help to elucidate whether its distribution is heterogeneous per se or a function of the effects of bipolar disorder and other factors such as concomitant medication use. Prediction of response to lithium is an important goal, and while ^7^Li-MRI will not be able to inform the decision to initiate lithium treatment, it holds the potential to identify patients most likely to benefit from remaining on lithium were responders to show reliably different distribution patterns to non-responders. The distribution of lithium might also relate to its effects on cognitive performance [46]—the interplay between GM, WM and orbital concentrations might aid our interpretation of lithium’s effects on functions such as processing speed, for example. Multimodal imaging studies combining conventional ^1^H MRI, ^7^Li-MRI and ^1^H-MRS techniques hold the potential to co-localise the action and concentration of lithium in the brain. Furthermore, there is potential to improve our ^7^Li-MRI technique through use of receive-array RF coils, complementary advanced scan methodologies such as compressed sensing and parallel imaging and the use of higher scanner field strengths.

Lithium is unusual in being a psychotropic drug and an element with exploitable MR properties—this has enabled the direct and non-invasive imaging of a major pharmaceutical agent in its target organ. The spatial distribution of lithium was imaged in the brains of subjects with bipolar disorder in a scan time of only 8 min. Our results show a spatially heterogeneous distribution of the ^7^Li-MR signal, which we believe reflects heterogeneity in lithium tissue concentration. Further planned work will elucidate whether individual differences in this heterogeneity are related to lithium treatment response.

## Electronic supplementary material

Supplemental material 1

## References

[1] Goodwin GM, Haddad PM, Ferrier IN, Aronson JK, Barnes TRH, Cipriani A (2016). Evidence-based guidelines for treating bipolar disorder: revised third edition recommendations from the British Association for Psychopharmacology. J Psychopharmacol.

[2] Malhi GS, Tanious M, Das P, Coulston CM, Berk M (2013). Potential mechanisms of action of lithium in bipolar disorder. CNS Drugs.

[3] Ramos P, Santos A, Pinto E, Pinto NR, Mendes R, Magalhaes T (2016). Alkali metals levels in the human brain tissue: anatomical region differences and age-related changes. J Trace Elem Med Biol.

[4] Moore GJ, Bebchuk JM, Parrish JK, Faulk MW, Arfken CL, Strahl-Bevacqua J (1999). Temporal dissociation between lithium-induced changes in frontal lobe myo-inositol and clinical response in manic-depressive illness. Am J Psychiatry.

[5] Lee JH, Adler C, Norris M, Chu WJ, Fugate EM, Strakowski SM (2012). 4-T ^7^Li 3D MR spectroscopy imaging in the brains of bipolar disorder subjects. Magn Reson Med.

[6] Renshaw PF, Wicklund S (1988). In vivo measurement of lithium in humans by nuclear magnetic resonance spectroscopy. Biol Psychiatry.

[7] Komoroski RA, Pearce JM, Newton JEO (1997). The distribution of lithium in rat brain and muscle in vivo by Li-7 NMR imaging. Magn Reson Med.

[8] Smith FE, Cousins DA, Thelwall PE, Ferrier IN, Blamire AM (2011). Quantitative lithium magnetic resonance spectroscopy in the normal human brain on a 3T clinical scanner. Magn Reson Med.

[9] Komoroski RA, Newton JEO, Sprigg JR, Cardwell D, Mohanakrishnan P, Karson CN (1993). In vivo 7Li nuclear magnetic resonance study of lithium pharmacokinetics and chemical shift imaging in psychiatric patients. Psychiatry Res.

[10] Girard F, Suhara T, Sassa T, Okubo Y, Obata T, Ikehira H (2001). ^7^Li 2D CSI of human brain on a clinical scanner. Magn Reson Mater Phys Biol Med.

[11] Soares JC, Boada F, Spencer S, Mallinger AG, Dippold CS, Wells KF (2001). Brain lithium concentrations in bipolar disorder patients: preliminary ^7^Li magnetic resonance studies at 3 T. Biol Psychiatry.

[12] Boada FE, Qian Y, Gildengers A, Phillips M, Kupfer D. In vivo 3D lithium MRI of the human brain. In: DK Sodickson, editor. Proceedings of the 18th Annual Meeting. International Society for Magnetic Resonance in Medicine: Stockholm, Sweden; 1st to 7th May 2010, p. 592.

[13] First MB, Spitzer RL, Gibbon M, Williams JBW (1997). Structured clinical interview for DSM-IV axis I disorders, research version.

[14] Hamilton M (1960). A rating scale for depression. J Neurol Neurosurg Psychiatry.

[15] Young RC, Biggs JT, Ziegler VE, Meyer DA (1978). A rating scale for mania: reliability, validity and sensitivity. Br J Psychiatry.

[16] Grof P, Duffy A, Cavazzoni P, Grof E, Garnham J, MacDougall M (2002). Is response to prophylactic lithium a familial trait?. J Clin Psychiatry.

[17] Haddad P, Wieck A, Yarrow M, Denham P (1999). The lithium side effects rating scale (LISERS): development of a self-rating instrument.

[18] Mann LW, Higgins DM, Peters CN, Cassidy S, Hodson KK, Coombs A (2016). Accelerating MR imaging liver steatosis measurement using combined compressed sensing and oarallel imaging: a quantitative evaluation. Radiology.

[19] Schneider CA, Rasband WS, Eliceiri KW (2012). NIH Image to ImageJ: 25 years of image analysis. Nat Methods.

[20] Jenkinson M, Beckmann CF, Behrens TE, Woolrich MW, Smith SM (2012). FSL. Neuroimage.

[21] Francis RI, Traill MA (1970). Lithium distribution in the brains of two manic patients. Lancet.

[22] Spirtes MA (1976). Lithium levels in monkey and human brain after chronic, therapeutic, oral dosage. Pharmacol Biochem Behav.

[23] Ebadi MS, Simmons VJ, Hendrickson MJ, Lacy PS (1974). Phamacokinetics of lithium and its regional distribution in rat-brain. Eur J Pharmacol.

[24] Ebara T, Smith DF (1979). Lithium levels in blood-platelets, serum, red-blood-cells and brain-regions in rats given acute or chronic lithium salt treatments. J Psychiatr Res.

[25] Edelfors S (1975). Distribution of sodium, potassium and lithium in brain of lithium-tretaed rats. Acta Pharmacol Toxicol (Copenh).

[26] Hanak AS, Chevillard L, El Balkhi S, Risede P, Peoc’h K, Megarbane B (2015). Study of blood and brain lithium pharmacokinetics in the rat according to three different modalities of poisoning. Toxicol Sci.

[27] Mukherjee BP, Bailey PT, Pradhan SN (1976). Temporal and regional differences in brain concentrations of lithium in rats. Psychopharmacol (Berl).

[28] Nelson SC, Herman MM, Bensch KG, Barchas JD (1980). Localization and quantitation of lithium in rat tissue following intra-peritoneal injections of lithium chloride. II. Brain. J Pharmacol Exp Ther.

[29] Rios C, Guzmanmendez R (1990). Determination of lithium in rat brain regions and synaptosomes by graphite furnace atomic absorption spectrophotometry. J Pharmacol Methods.

[30] Smith DF, Amdisen A (1981). Lithium distribution in rat-brain after long-term central administration by minipump. J Pharm Pharmacol.

[31] Thellier M, Heurteaux C, Wissocq JC (1980). Quantitative study of the distribution of lithium in the mouse-brain for various doses of lithium given to the animal. Brain Res.

[32] Davenport VD (1950). Distribution of parenterally administered lithium in plasma, brain and muscles of rats. Am J Physiol.

[33] Ho AKS, Gershon S, Pinckney L (1970). Effects of acute and prolongued lithium treatment on distribution of electrolytes, potassium and sodium. Arch Int Pharmacodyn Ther.

[34] Ozawa H, Nozu T, Aihara H, Akiyama F, Sasajima M (1976). Metabolic fate of lithium salts by single and repeated adminstration and behavioral effects. Folia Pharmacol Jpn.

[35] Zanni G, Michno W, Di Martino E, Tjarnlund-Wolf A, Pettersson J, Mason CE (2017). Lithium accumulates in neurogenic brain regions as revealed by high resolution ion imaging. Sci Rep.

[36] Ramaprasad S, Newton JEO, Cardwell D, Fowler AH, Komoroski RA (1992). In vivo ^7^Li NMR imaging and localized spectroscopy of rat brain. Magn Reson Med.

[37] Ramaprasad S (2004). Lithium spectroscopic imaging of rat brain at therapeutic doses. Magn Reson Imaging.

[38] Komoroski RA (2005). Biomedical applications of Li-7 NMR. NMR Biomed.

[39] Ramaprasad S (2005). Magnetic resonance spectroscopic imaging studies of lithium. Progress Nucl Magn Reson Spectrosc.

[40] Ramaprasad S, Ripp E, Pi JX, Lyon M (2005). Pharmacokinetics of lithium in rat brain regions by spectroscopic imaging. Magn Reson Imaging.

[41] Stout J, Hanak A-S, Chevillard L, Djemai B, Risede P, Giacomini E et al. Investigation of lithium distribution in the rat brain *ex vivo* using lithium-7 magnetic resonance spectroscopy and imaging at 17.2T. *NMR Biomed* 2017;30:e3770. 10.1002/nbm.3770 28703506

[42] Werstiuk ES, Seggie J, Joshi M (1984). Lithium in pigmented eye rats: effects of dose and time of day on drinking, body weight, retinal and blood distribution. Progress Neuropsychopharmacol Biol Psychiatry.

[43] Gyulai L, Wicklund SW, Greenstein R, Bauer MS, Ciccione P, Whybrow PC (1991). Measurement of tissue lithium concentration by lithium magnetic-resonance spectroscopy in patients with bipolar disorder. Biol Psychiatry.

[44] Kato T, Inubushi T, Takahashi S (1994). Relationship of lithium concentrations in the brain measured by 7Li magnetic resonance spectroscopy to treatment response in mania. J Clin Psychopharmacol.

[45] Kato T, Fujii K, Shioiri T, Inubushi T, Takahashi S (1996). Lithium side effects in relation to brain lithium concentration measured by lithium-7 magnetic resonance spectroscopy. Prog Neuropsychopharmacol Biol Psychiatry.

[46] Wingo AP, Wingo TS, Harvey PD, Baldessarini RJ (2009). Effects of lithium on cognitive performance: a meta-analysis. J Clin Psychiatry.

