# Supplementary material for: 3D 7Li magnetic resonance imaging of brain lithium distribution in bipolar disorder
Source: Mol Psychiatry. 2018 Feb 9;23(11):2184–91. doi: 10.1038/s41380-018-0016-6 (PMC5955212; doi:10.1038/s41380-018-0016-6)
Supplement: Supplementary file 1 — Supplemental material 1 [file 41380_2018_16_MOESM1_ESM.docx]

**SUPPLEMENTARY INFORMATION**

**Methods**

*Optimisation of ^7^Li 3D balanced steady state free precession (b-SSFP) and ^7^Li 3D spoiled gradient echo (SPGR) imaging sequences*

The in vivo T_1_ of ^7^Li was measured in the eight subjects who underwent ^7^Li b-SSFP MRI using a non-localised inversion-recovery sequence. Spectra were acquired into 2048 data points (spectral width: 8 kHz) at five inversion times (1, 2.5, 5, 7.5 and 10 s), with five averages per spectrum. The repetition time (TR) was 10.3 s. Lithium T_1_ was determined by fitting Equation 1 to ^7^Li signal amplitude, where *S(t)* represents the T_1_-dependent variation in ^7^Li peak amplitude with inversion time *t*.

$$S(t)=S_{0}\left[ 1-2e^{-\frac{t}{T_{1}}} \right]$$

*Equation 1*

In the four subjects who underwent the extended scanning protocol, ^7^Li SPGR images were acquired with Ernst angle excitation,^43^ calculated based on the in vivo T_1_ and the minimum achievable TR for the pulse sequence.

*^7^Li flip angle calibration*

Correct radiofrequency (RF) power calibration was confirmed on a per-subject basis with a ^7^Li flip angle calibration. Non-localised ^7^Li spectra were acquired at six nominal flip angles between 30 and 180 degrees (TR = 10 s, five averages per spectrum). A sinusoidal function was fitted to spectral peak amplitudes to determine discrepancy between requested and achieved flip angle.

*Modelling of ^7^Li b-SSFP signal intensity versus ^7^Li SPGR*

The dependence of ^7^Li amplitude signal on T_1_ and T_2_ relaxation times was calculated using the b-SSFP steady state signal equation^44^ shown in Equation 2. This equation is valid in the regime where TR (9.4 ms in our experiments) << T_1_, T_2_.

$$S_{bSSFP}=\frac{\rho\cdot\sin\left( \alpha\right)}{\left( 1+\frac{T_{1}}{T_{2}} \right)+\left( 1-\frac{T_{1}}{T_{2}} \right)\cos\left( \alpha\right)}$$

*Equation 2*

Similarly, the relaxation time dependence of signal in the spoiled gradient echo sequence was calculated using Equation 3.

$$S_{SPGR}=\frac{\rho\cdot\sin\left( \alpha\right)\left( 1-e^{-TR/T_{1}} \right)e^{TE/T_{2}}}{\left( 1-{\cos\left( \alpha\right)e}^{-TR/T_{1}} \right)}$$

*Equation 3*

In both cases, α is flip angle, ρ is spin density (constant in our calculations), T_1_ and T_2_ are the lithium relaxation times, TE is the sequence echo time and TR is the repetition time. Lithium T_2_ was set at 150 ms, which represents an estimate centered on the wide range of in vivo values reported in the literature.^37, 45, 46^

Relative signal amplitude between the b-SSFP and SPGR was calculated for T_1_ values between 2 and 10 s using the selected flip angles (60 degrees for b-SSFP and 4.6 degrees for SPGR).

**Results**

*^7^Li flip angle optimisation*

Calibration of ^7^Li RF power requirements demonstrated a discrepancy of up to 20% between requested and achieved flip angle for five out of eight subjects due to differences in coil loading. Correct ^7^Li flip angle was achieved in b-SSFP and SPGR image acquisitions by manual adjustment of requested flip angle at the scanner interface.

*^7^Li flip angle calibration*

Optimal flip angle for ^7^Li b-SSFP imaging was calculated as 60 degrees for TR = 9.3 ms and TE = 4.5 ms respectively. Optimal flip angle for ^7^Li SPGR imaging for a TR of 6.7 ms and a T_1_ of 2.1 s (previous measurements^7^) was calculated as 4.6 degrees.

*^7^Li T_1_ measurement*

The T_1_ of in vivo ^7^Li was 2.3 ± 0.6 ms in the eight subjects studied, not significantly different to our previous assessment of 2.1 s.^7^

*Modelling of ^7^Li b-SSFP signal intensity versus ^7^Li SPGR*

Modelling using equations 2 and 3 showed that the ^7^Li b-SSFP sequence would be expected to achieve a signal to noise ratio (SNR) benefit of 2.1 × relative to that achieved using the SPGR method for lithium with a T_1_ of 2.1 s as measured in our subjects and assuming T_2_ of 0.15 s. The enhancement factor varied by less than 30% across the 2 to 10 s T_1_ range investigated, indicating that the ^7^Li b-SSFP sequence would not be expected to exhibit major variation in signal due to differences in T_1_.
